# Gene expression profiling identifies tumour markers potentially playing a role in uveal melanoma development

**DOI:** 10.1038/sj.bjc.6601374

**Published:** 2003-11-11

**Authors:** W Zuidervaart, P A van der Velden, M H Hurks, F A van Nieuwpoort, C J J Out-Luiting, A D Singh, R R Frants, M J Jager, N A Gruis

**Affiliations:** 1 Department of Ophthalmology, Leiden University Medical Centre, Postbus 9600, 2300 RC Leiden, The Netherlands; 2 Department of Dermatology, Leiden University Medical Centre, Leiden, The Netherlands; 3 Department of Ophthalmology, Royal Hallamshire Hospital, Sheffield, UK; 4 Department of Human Genetics, Leiden University Medical Centre, Leiden, The Netherlands

**Keywords:** uveal melanoma, gene expression, microarray

## Abstract

Microarray is a powerful tool to compare the gene expression of different tumour specimens and cell lines simultaneously and quantitatively. To get a better insight into genes that are involved in uveal melanoma tumorigenesis, we compared the gene expression profiles of 12 different uveal melanoma cell lines with three melanocyte cell cultures obtained from healthy donor eyes. Gene expression profiles were obtained by nylon filter arrays, containing 1176 gene spots related to cancer development. The expression levels of selected genes were validated on cell lines and primary uveal melanomas by real time RT–PCR, and were subsequently included in cluster analysis. Four candidate tumour markers, Laminin Receptor 1, Endothelin 2, Von Hippel Lindau Binding protein 1 and Cullin 2, have been selected from genes that were differentially expressed in the uveal melanoma cell lines compared to the normal uveal melanocytes. In primary uveal melanomas, these four markers could discriminate between two classes of uveal melanoma, which may be indicative of a differential disease process.

Up- and downregulated genes can be important determinants of tumour development. To understand the biological behaviour of tumour growth and progression, it is important to know how genes are expressed in malignancies, compared to normal tissue.

Uveal melanoma is the most common primary intraocular tumour of the eye in adults. The incidence ranges from six to eight cases per one million subjects per year among Caucasians. Approximately, 50% of patients with large uveal melanoma die from metastases (Diener-West *et al*, 1992).

The well-known prognostic parameters for uveal melanoma are clinico-histopathological parameters cell type, tumour diameter, tumour localisation, mitotic rate and the presence of vascular networks (Mooy and de Jong, 1996). Other defined factors are, for instance, the expression of the epidermal growth factor receptor (Hurks *et al*, 2000) and the reduced CDKN2A expression caused by promoter hypermethylation (van der Velden *et al*, 2001). In spite of these markers, metastases can still not be detected in an early stage. The mortality rates, after metastasis has occurred, are very high and hardly any treatment is effective for uveal melanoma metastases. Therefore, better insight into tumour progression and tumour dissemination is still of utmost importance.

Information regarding genes that are involved in the tumour growth of uveal melanoma and in the development of metastases is frequently derived from studies that relate biological parameters of tumour progression to prognostic outcome. For instance, loss of chromosome 3 and gain of the q arm of chromosome 8 are significantly associated with poor survival and loss of 1p is a specific hallmark of primary uveal melanoma metastases (White *et al*, 1998; Aalto *et al*, 2001).

Recently, cDNA microarray studies have become a powerful tool to detect differences in gene expression in different kinds of tumours (Ross *et al*, 2000), and have subsequently demonstrated that expression profiling can serve as a prognostic tool (Alizadeh *et al*, 2001; van de Vijver *et al*, 2002).

In this study, we used filter arrays with more than a thousand cDNAs related to cancer development, to reveal genes that are differentially expressed in uveal melanoma cell lines compared to cultured normal human melanocytes. In order to validate potential progression markers, real-time quantitative RT–PCR analysis on differentially expressed genes was used in cell lines and in fresh frozen primary uveal melanoma specimens.

An important role in uveal melanoma development is now suggested for Laminin Receptor 1 (LAMR1), Endothelin 2 (ET2), Von Hippel Lindau Binding Protein 1 (VBP1) and Cullin 2 (CUL2) genes, since their expression levels discriminate between two classes of uveal melanoma.

## MATERIALS AND METHODS

### Cell lines and tumour characteristics

A total of 15 cell lines were included in this study. Nine cell lines were derived from primary choroidal uveal melanomas (Mel202, Mel285, Mel270, Mel290, 92.1, 92.2, OCM1, OCM3 and OCM8), three cell lines were derived from uveal melanoma metastases (Omm1.3 and Omm1.5 are liver metastases from Mel270, and OMM1 was obtained from a subcutaneous metastasis) (Luyten *et al*, 1996). The cell lines OCM1, OCM3 and OCM8 were kindly provided by Dr Kan-Mitchell (Kan-Mitchell *et al*, 1989). Cell lines Mel202, Mel285, Mel290, Mel270 and the two cell lines derived from liver metastases were provided by Professor BR Ksander (Schepens Eye Institute, Boston, MA, USA). Cell lines 92.1 and 92.2 were derived from the same primary tumour and were established in our own laboratory (Waard-Siebinga *et al*, 1995).

Three melanocyte cell cultures were grown from three normal human donor eyes of two different individuals (Melcyt1A, Melcyt1B and Melcyt2). After the corneae of these eyes were prepared for donor purposes, the remaining tissues were kindly provided by the Netherlands Ophthalmic Research Institute (Eye Bank, IOI, Amsterdam). The freshly obtained eyes were rinsed with PBS and superfluous tissue was removed. The eye was cut into two parts, and the part containing the iris and lens was discarded. The vitreous body and retina of the remaining part were removed. After incubation in 0.125% trypsin (Gibco, Paisley, Scotland) in 0.02% EDTA during 1 h at 37°C the retinal pigment epithelium was removed. The choroid was peeled off the sclera and incubated in 0.25% trypsin during 18 h at 4°C. After the addition of culture medium, the cells were collected by centrifugation and plated. Geneticin (Gibco) was added to the culture medium to a final concentration of 100 *μ*g ml^−1^ to eliminate contaminating cells, like fibroblasts. The cell cultures were characterised by immunohistochemistry for S100 and NKI/beteb (Vennegoor *et al*, 1988).

The melanoma cell lines were cultured in RPMI 1640 (Gibco) medium and supplemented with 3 mM L-glutamine (Gibco), 2% penicillin/streptomycin and 10% FBS (Hyclone, UT). The three melanocyte cell lines were grown in F12 medium (Gibco), following the directions of Hu *et al* (1993). All cell cultures were incubated at 37°C in a humidified 5% CO_2_ atmosphere.

The fresh frozen uveal melanoma samples were derived from 19 different primary choroidal melanomas. The research protocol followed the Tenets of the Declaration of Helsinki. Histopathological analysis was performed by an ocular pathologist. Table 1

**Table 1:** Clinical and pathological characteristics of 19 fresh frozen primary uveal melanoma samples

**Tumour sample**	**Cell type**	**Prominence (mm)**	**Diameter (mm)**	**Pigment**	**Mitosis^a^**	**Intravascular ingrowth**	**Vascular loops**	**Metastases**	**Location**
1	Epithelioid	8	14	>50%	3	Yes	Yes	No	Chor^b^
2	Spindle	10	12	>50%	2	No	Yes	No	Chor
3	Mixed	7	15	>50%	3	No	Yes	Yes	Cbd-chor^c^
4	Spindle	11	11	<50%	5	No	Yes	No	Cbd-chor
5	Mixed	9	14	>50%	5	Yes	No	No	Cbd-chor
6	Mixed	12	17	<50%	8	No	Yes	No	Chor
7	Spindle	9	16	<50%	2	No	Yes	No	Cbd-chor
8	Mixed	8	14	Severe	NA^d^	No	Yes	No	Chor
9	Mixed	8	17	<50%	5	Yes	Yes	No	Cbd-chor
10	Mixed	9	16	>50%	4	No	Yes	No	Cbd-chor
11	Mixed	13	20	>50%	7	No	Yes	No	Cbd
12	Spindle	11	20	>50%	20	No	Yes	No	Cbd
13	Mixed	13	18	50%	21	Yes	Yes	No	Cbd-chor
14	Mixed	13	20	<50%	20	No	NA	No	Chor
15	Mixed	6	18	>50%	1	No	No	No	Cbd-chor
16	Mixed	11	20	50%	21	Yes	No	No	Cbd-chor
17	Mixed	8	21	<50%	6	No	Yes	No	Chor
18	Mixed	8	20	Severe	9	Yes	Yes	No	Cbd-chor
19	Mixed	12	20	50%	10	No	Yes	No	Cbd-chor

a Number of mitoses per 15 fields of microscopic examination at a magnification of × 320.

b Chor=choroidea.

c Cbd=ciliar body.

d NA=not available.

presents the clinical and pathological characteristics of the fresh frozen uveal melanoma samples. We are limited in survival analysis due to follow-up data of three years.

### Microarray cDNA hybridisation and analysis

Cells from early passages were grown to subconfluency and the total RNA of each cell line was isolated with the RNeasy kit, following the directions of the manufacturer (Qiagen, Hilden, Germany). Biotin-labelled oligo-dT and streptavidin-labelled magnetic beads were used to isolate the mRNA from the total RNA. Probe synthesis and hybridisation were performed according to the protocol of the manufacturer of the Human Cancer Atlas 1.2 filters (Clontech, Palo Alto, CA, USA). The cDNA was labelled with *α*^32^P-dATP. Each cell line was hybridised to two separate filters in order to obtain a technical replicate. In this study, the melanocyte cell lines were used as references and controls. Cell lines Melcyt1A and Melcyt1B are derived from two different eyes of one person in order to create an internal biological control.

A nylon Human Cancer Atlas filter consists of 1176 different cDNA spots designed to reflect molecular pathways in oncogenesis and several house-keeping genes.

To analyse and quantify the spots obtained by the phosphorimager scans, we used the GenePix® Pro analyzing computer program (Axon Instruments, Inc., Union City, CA, USA). Each cell line was normalised to the value of the uveal melanocyte, Melcyt1A. The obtained ratios were analysed by a hierarchical clustering method (Cluster) to compare the expression data. In this type of clustering method, the data points are forced into a strict hierarchy of nested subsets, resulting in a phylogenetic tree with branch lengths that indicate the degree of similarity between the samples (Tamayo *et al*, 1999). The data obtained by these analysis programs were visualised by TreeView (Eisen *et al*, 1998; Tavazoie *et al*, 1999).

### Real-time quantitative RT–PCR analysis

To validate the results of the microarray, we performed real-time quantitative RT–PCR analyses on the cell lines and primary frozen tumour samples (Heid *et al*, 1996). The same method and protocol of the RNeasy kit was applied to isolate total RNA from sections of the fresh frozen melanoma samples. In all 1 *μ*g of total RNA of all cell lines and tumour samples was reverse-transcribed with M-MLV reverse transcriptase (Gibco-BRL, Eggenstein, Germany) in a 20 *μ*l reaction volume. The cDNA synthesis was performed in the presence of random hexamere primers at a temperature of 42°C for 50 min (Promega, San Louis Obispo, CA, USA). We applied the real-time PCR method using the SYBR® Green PCR Master Mix as supplied by the manufacturer (PE Applied Biosystems, Inc., Foster City, CA, USA). The SYBR® Green I dye detects double-stranded DNA, not requiring specific probes. The Mix includes a passive reference, which is required for signal normalisation. To minimise nonspecific product formation, the reaction Master Mix contains the enzyme AmpliTaq Gold® DNA polymerase. An ABI PRISM 7700 Sequence Detection System monitors the fluorescence signal during the PCR reaction. It registers the number of cycles at which the signal reaches a certain threshold level. The differences between the threshold value of the target transcripts and the endogenous references are used to calculate the relative expression.

The primers for the four genes under study, ET2, LAMR1, VBP1 and CUL2, and the two endogenous controls U1A and RPS11 were designed with the Primer Express software (PE Applied Biosystems) (Table 2

**Table 2:** Primer sequences of the genes validated in the real-time quantitative RT–PCR assays

**Primer**	**Primer (forward)**	**Primer (reverse)**
U1A	5′-gca gct tat gcc agg aca gat-3′	5′-ttg gtg agg aac aag atg tga ttc-3′
RPS11	5′-aag cag ccg acc atc ttt ca-3′	5′-cgg gag ctt ctc ctt gcc-3′
ET2	5′-tgg agg ctg cgt cct ga-3′	5′-ctg agg cat gcg tgt tcg t-3′
LAMR1	5′-cca ctg gag cca ctc caa tt-3′	5′-ggc tgc ctg gat ctg gtt agt-3′
VBP-1	5′-gac cag att ctt gct ggc aga t-3′	5′-agc ccc caa cca cag aca c-3′
CUL-2	5′-gtt cgt atc atg aaa gca cga aaa-3′	5′-tta aac cta gct ctt gac tgg cta atc-3′

). All reactions were repeated twice with two different endogenous controls to ensure reproducibility of the results. For comparing the efficiency of amplification of the gene targets and the controls, a serial dilution was made of each different cDNA sample. All experiments revealed a nearly optimal efficiency close to 100%.

### Statistical analysis

Correlations between clinico-histopathological parameters and the expression level of the four genes were determined using the Pearson correlation test.

## RESULTS

### Microarray analysis

In this study, the expression profile of 12 different human uveal melanoma cell lines was compared to three uveal melanocyte cultures. The mRNA of all cell cultures was hybridised in replicate to filter arrays containing 1176 cDNAs related to tumorigenesis. The duplicate experiments revealed nearly identical hybridisations that clearly showed the fingerprints of each cell line. Nevertheless, a small percentage of genes were differentially expressed between the duplicates. Only the few genes with opposite results were discarded from analysis. The gene expression of each cell line was normalised to one melanocyte cell culture (Melcyt1A). A total of 449 genes revealed no expression in all 15 cell lines, that is, their expression was not significantly higher than the local background. Only 52 genes were expressed in all cell cultures, that is, melanomas and melanocytes. In total, 54 genes were expressed only in melanoma cell lines and 187 genes exclusively in the melanocytes.

In at least eight melanoma cell lines, 29 genes were expressed that were not expressed in any of the three normal melanocyte cultures (not significantly higher than the local background). Fifteen of these genes were highly differentially expressed, that is, we considered a gene to be highly expressed if the expression ratio of an uveal melanoma cell line was at least 1.5-fold higher compared to the normal uveal melanocytes Melcyt 1B and Melcyt 2 (Table 3

**Table 3:** Microarray results: selection of genes revealing high differential expression ratios in uveal melanoma cell lines normalised to uveal melanocyte 1A (Melcyt1A)

**Name**	**Melcyt1B**	**Melcyt2**	**Mel270**	**Omm1.3**	**Omm1.5**	**Mel202**	**Mel285**	**Mel290**	**92.1**	**92.2**	**Ocm1**	**Ocm3**	**Ocm8**	**Omm1**
LAMR1	0.87	1.11	3.62	6.66	4.72	2.15	2.40	2.28	7.24	6.33	6.30	3.04	3.21	4.47
ET2	1.18	1.39	26.68	34.92	42.63	1.18	0.92	1.34	3.67	3.47	50.11	8.32	4.02	36.48
CUL2	1.16	1.15	1.05	1.14	1.17	1.69	1.81	3.69	3.82	4.35	10.27	6.53	1.47	6.90
VBP1	1.09	1.42	0.89	0.94	1.01	2.19	3.52	9.64	6.52	4.75	3.87	3.23	2.02	2.64
RHOC	1.13	1.28	2.66	3.77	3.27	1.47	1.48	1.62	2.25	2.41	4.43	3.08	3.58	3.18
PKCI1	1.21	1.46	5.1	3.28	1.71	2.41	3.34	5.16	8.3	5.71	4.32	3.43	3.55	4.85
TIMP1	0.83	0.96	3.03	9.90	15.24	1.07	2.20	1.80	2.01	1.85	4.05	2.96	2.82	4.06
SOD1	0.89	1.06	2.21	0.84	0.65	1.94	3.30	2.48	5.40	5.73	3.16	1.91	2.94	2.01
HMGIY	0.89	0.78	3.93	1.95	0.81	1.62	2.92	1.18	2.47	2.04	2.27	1.35	1.52	1.79
RPS5	0.76	0.80	3.18	6.01	3.51	1.58	1.77	1.09	4.50	2.79	2.13	1.44	2.33	1.22
CCND1	0.89	1.06	1.39	0.85	1.07	2.35	3.52	1.69	2.37	1.84	2.12	3.40	3.35	1.69
IL13	1.19	1.17	5.19	2.70	2.92	1.22	0.99	0.93	3.01	4.08	177.83	17.15	6.01	24.37
EIF3-beta; EIF3B	0.99	0.73	2.71	1.15	1.06	2.80	2.87	1.55	1.85	5.01	1.60	1.27	1.83	1.14
ETV4; E1A-F	1.17	0.82	2.20	1.25	1.17	1.54	0.92	1.00	1.94	2.21	34.47	7.70	24.75	5.99
CDC37 homolog	0.99	0.92	2.91	1.38	1.16	2.01	2.05	1.39	3.81	4.35	2.98	1.56	1.53	1.44

). From this list, we selected four genes revealing the highest percentage of uveal melanoma cell lines displaying an increase in the expression ratio compared to the controls (Table 3).

Furthermore, the common origin of the primary tumour cell line Mel 270 and its two liver metastatic cell lines Omm1.3 and Omm1.5 was perfectly demonstrated by their similar expression profiles (Table 3). Hierarchical cluster analysis revealed their close relationship in a phylogenetic cluster tree. The similarity could be clearly recognised in the tree by the short branches reflecting their degree of correlation. The latter was also apparent for cell lines 92.1 and 92.2, both derived from the same primary tumour. The genetic origin of these cell lines appears to be the major determinant of expression, since the lines resemble each other more than any of the other cell lines, indicating that these cell lines have kept their identity during *in vitro* culturing and their reliability as a model for tumour research. This importance of genetics in gene expression was also demonstrated by Cheung *et al* (2003).

### Real-time quantitative PCR analysis

#### Cell lines

ET2, LAMR1, VBP1 and CUL2 belong to the group of genes exhibiting the most discriminating differential expression ratio in uveal melanomas cell lines compared to the expression ratio of the normal melanocytes (Table 3). We validated the differential expression of these genes by real-time RT–PCR. Similar expression of the two endogenous controls U1A (small nuclear protein) and RPS11 (ribosomal protein) excluded possible influences of regulation.

The absolute expression levels of the four genes were markedly different. The average expression of CUL2 was 64 times higher compared to the average expression level of ET2, VBP1 showed 512 times higher and LAMR1 4096 times higher than ET2.

Despite differences in absolute expression levels obtained by filter array and real time RT–PCR, both assays separately confirmed the importance of these genes.

Comparing the expression data between the filter array and the real-time PCR, real time RT–PCR confirmed a high expression level of ET2 in two of the 12 cell lines. The VBP1 gene revealed a high expression in nine out of 12 cell lines *vs* seven out of 12 cell lines in the PCR assay, with corresponding results between both assays in the case of four cell lines (Mel285, Ocm3, Ocm8 and 92.1). The same trend was observed in the expression pattern of CUL2; in the microarray eight out of 12 cell lines revealed a high expression compared to normal melanocyte expression, whereas in the PCR assay nine out of 12 cell lines fulfilled this criterion.

LAMR1 expression in the microarray was very consistent with its expression measured by real-time PCR. In both assays we observed low expression in the melanocytes and cell lines Ocm8, Mel202 and Mel290, whereas the cell lines 92.1, Ocm1, Omm1 and Omm1.3 revealed high expression levels. This is of great interest, since two of these cell lines with a high expression level of LAMR1 are derived from metastases.

### Fresh frozen primary uveal melanoma samples

In order to validate the candidate tumour markers obtained in cell lines, we performed real-time quantitative PCR analysis on 19 fresh frozen uveal melanoma samples.

The genes LAMR1 and especially ET2 revealed highly differential expression levels, whereas the expression levels of the genes CUL2 and VBP1 were not markedly highly differential.

**Figure 1:**
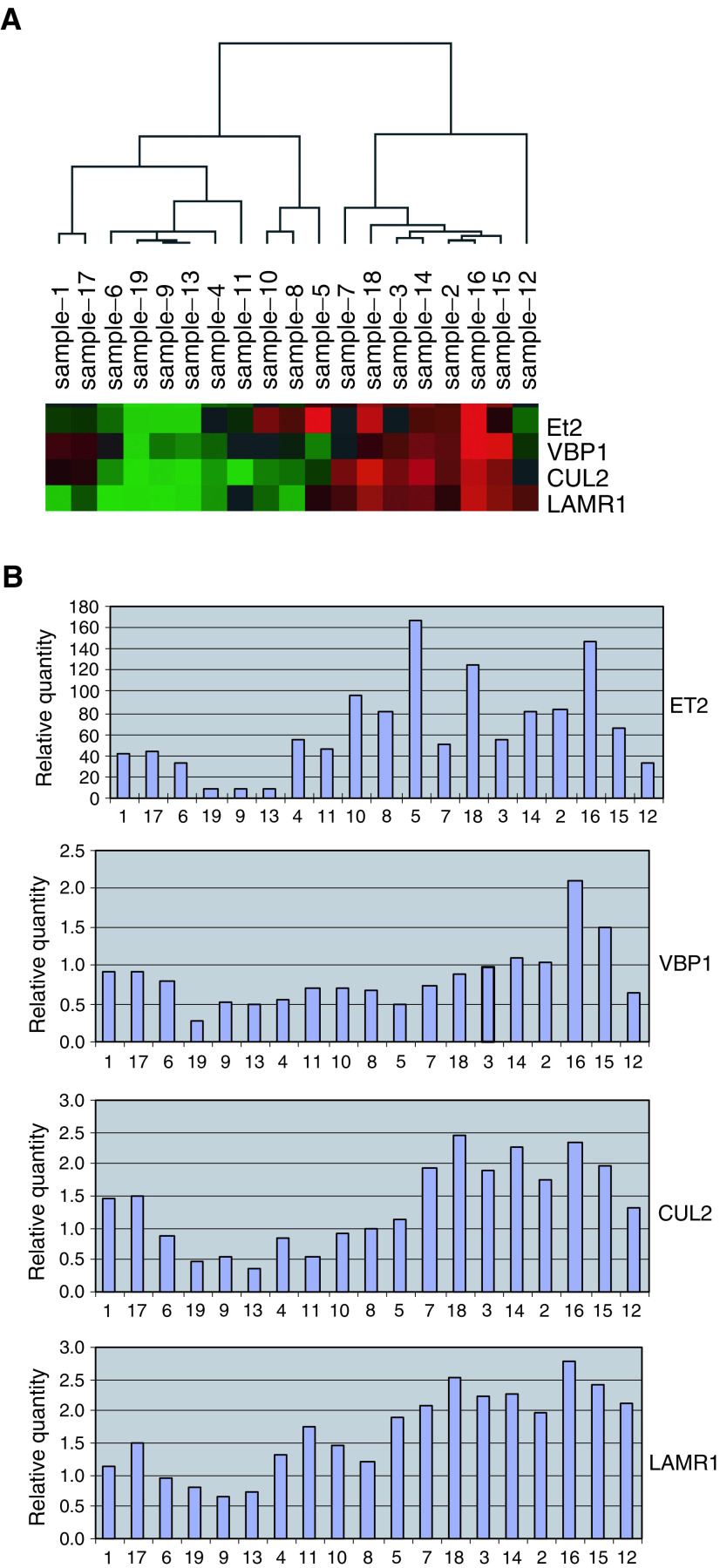
(**A**) Cluster figure (Treeview) of the real-time RT–PCR results of fresh frozen uveal melanoma samples. Red indicates a relatively high expression level and green indicates a relative low expression level. (**B**) Column graphs presenting the relatively quantity of expression per gene. The samples are set in the same order as in the cluster figure. The differences in the relative expression levels between the two clusters of the uveal melanoma samples are demonstrated in these graphs as well.

Cluster analysis of the real-time PCR results of the genes under study revealed two distinct clusters in the primary tumour samples (Figure 1

). The first cluster was formed by eight samples revealing a relatively high expression of the genes ET2, LAMR1, CUL2 and VBP1. The relatively low expression levels of all these genes were observed in a second cluster of 11 tumour samples.

Pearson test identified one significant correlation between gene expression and histopathological parameters of the tumours; ET2, LAMR1 and VBP1 were positively correlated with PAS-positive loops (*P*=0.005, 0.028 and 0.01, respectively).

We were not able to perform a solid survival analysis, since all tumours were collected recently.

## DISCUSSION

We used microarray technology and real-time RT–PCR to compare gene expression profiles of uveal melanoma cell lines and normal melanocytes, in order to identify potential tumour progression markers.

Based on the type and purpose of our array technique, we validated a selection of four genes, that is, LAMR1, ET2, VBP1 and CUL2, with high differential expression by quantitative real-time RT–PCR. In addition, we performed real-time RT–PCR of these four genes in a panel of primary uveal melanomas. Hierarchical clustering of these data resulted in two distinct subgroups with differences in the expression level.

Between the two assays, there is a difference in expression level of the selected genes. The higher the average expression level, the stronger the correlation with the microarray results. The filter technique applied in this study was not able to detect genes with a low expression level securely enough to obtain a valid estimate of the expression differences; nevertheless, it appeared to be a useful method to identify a combination of genes that might play a role in uveal melanoma development.

The expression patterns of the genes VBP1 and CUL2 exhibited a small overlap in both assays and revealed no elevated expression levels in the primary tumour samples. Both genes are Von Hippel-Lindau (VHL)-related genes. VHL regulates transcription elongation and is known to act as a tumour suppressor protein. CUL2 is a member of the cullin family of proteins and binds to the VHL/elongin B/elongin C complex (VBC). The members are believed to target certain cellular proteins for ubiquitination and degradation and are in some cases involved in cell cycle regulation (Pause *et al*, 1997; Clifford *et al*, 1999). VBP1 forms complexes with the VHL gene product. VHL acts as a molecular chaperone that carries VBP1 from perinuclear granules to the nucleus or cytoplasm (Tsuchiya *et al*, 1996). In spite of the highly differential expression ratios of these two genes in the microarray, the expression levels in the primary uveal melanomas were not high. Nevertheless, they seem to have a discriminating role in the primary uveal melanomas.

The microarray data displayed differential expression of ET2 in nine of the 12 cell lines. However, real-time PCR confirmed an upregulated ET2 expression in only two of the 12 melanoma cell lines. On the other hand, all 19 tumour samples revealed a high expression of ET2. The validation of ET2 in the cell lines would not consider this gene to be of importance for uveal melanoma development. However, several studies provided abundant evidence for ET2 in cancer development. The NCI60 Cancer Microarray project described a high expression level of the endothelin receptor type B (ENRB) in skin melanoma (Ross *et al*, 2000), and Demunter *et al* (2001) reported evidence for its role as a tumour progression marker in this tumour type.

ENRB is a nonspecific receptor for all three isotypes of endothelin: ET1, ET2 and ET3 (Arai *et al*, 1990; Sakurai *et al*, 1990). ET2 can act as a mitogen in many cancer systems, and ETs in general stimulate melanocyte proliferation and differentiation (Reid *et al*, 1996). Recently, Smith *et al* (2002) associated decreased ENRB expression in large primary uveal melanoma with early clinical metastasis and short survival. These results, together with the high expression levels of ET2 in our tumour samples, might support investigation of the expression of endothelin receptors and their ligands in uveal melanoma.

LAMR1 revealed the most interesting results. Combining both assays we observed at least a 2.5-fold higher LAMR1 expression ratio in eight uveal melanoma cell lines (including all metastatic cell lines Omm1.3, Omm1.5 and Omm1) compared to the basic expression ratio of normal melanocytes. Furthermore, eight of the 19 primary tumour samples revealed relatively high expression levels of LAMR1, including the only patient in whom metastases had already developed. Interestingly, LAMR1 is significantly correlated with the PAS-positive loops, containing laminin (Thies *et al*, 2001). LAMR1 is known for its role in cancer proliferation, invasion and metastasis (Satoh *et al*, 1992; Castronovo, 1993). Hipfel *et al* (2000) observed a high expression of this laminin receptor in skin melanoma but not in naevi and normal skin. Despite the lack of survival data in our study, an interesting relationship between the level of LAMR1 expression and metastatic potential might be present, encouraging further exploration of the role of LAMR1 in the development of uveal melanoma metastases.

Validation of the four genes on the primary melanoma samples resulted in two distinct subgroups with specific expression profiles. Besides LAMR1, the histopathologic parameter PAS-positive loops revealed a significant association with the expression level of the genes ET2 and VBP1. The ability to form vascular loops is an important prognostic parameter in uveal melanoma metastases development (Folberg *et al*, 2001). Therefore, these two clusters within these samples might have a prognostic value in tumour dissemination.

In conclusion, filter microarray is a strong tool to compare the gene expression profiles of cell lines of different origin, but careful attention should be paid to the quantitative value of this filter method in the case of genes with low expression levels. By dint of this powerful technique, we identified discriminating fingerprints, and subsequently defined tumour markers possibly involved in uveal melanoma tumour development and progression, such as ET2 and LAMR1.
